# Cyclodextrin inhibits zinc corrosion by destabilizing point defect formation in the oxide layer

**DOI:** 10.3762/bjnano.9.86

**Published:** 2018-03-20

**Authors:** Abdulrahman Altin, Maciej Krzywiecki, Adnan Sarfraz, Cigdem Toparli, Claudius Laska, Philipp Kerger, Aleksandar Zeradjanin, Karl J J Mayrhofer, Michael Rohwerder, Andreas Erbe

**Affiliations:** 1 Max-Planck-Institut für Eisenforschung GmbH, Max-Planck-Str. 1, 40237 Düsseldorf, Germany; 2 Institute of Physics–CSE, Silesian University of Technology, Konarskiego 22B, 44-100 Gliwice, Poland; 3 Forschungszentrum Jülich GmbH, Helmholtz Institute Erlangen-Nürnberg for Renewable Energy (IEK-11), Egerlandstraße 3, 91058 Erlangen, Germany; 4 Department of Materials Science and Engineering, NTNU, Norwegian University of Science and Technology, 7491 Trondheim, Norway

**Keywords:** band diagram, defect chemistry, organic corrosion inhibitors, X-ray photoelectron spectroscopy, zinc corrosion

## Abstract

Corrosion inhibitors are added in low concentrations to corrosive solutions for reducing the corrosion rate of a metallic material. Their mechanism of action is typically the blocking of free metal surface by adsorption, thus slowing down dissolution. This work uses electrochemical impedance spectroscopy to show the cyclic oligosaccharide β-cyclodextrin (β-CD) to inhibit corrosion of zinc in 0.1M chloride with an inhibition efficiency of up to 85%. Only a monomolecular adsorption layer of β-CD is present on the surface of the oxide covered metal, with Raman spectra of the interface proving the adsorption of the intact β-CD. Angular dependent X-ray photoelectron spectroscopy (ADXPS) and ultraviolet photoelectron spectroscopy (UPS) were used to extract a band-like diagram of the β-CD/ZnO interface, showing a large energy level shift at the interface, closely resembling the energy level alignment in an n–p junction. The energy level shift is too large to permit further electron transfer through the layer, inhibiting corrosion. Adsorption hence changes the defect density in the protecting ZnO layer. This mechanism of corrosion inhibition shows that affecting the defect chemistry of passivating films by molecular inhibitors maybe a viable strategy to control corrosion of metals.

## Introduction

Organic corrosion inhibitors are usually described to work by limiting anodic and/or cathodic processes, either by adsorptive blocking of active sites, or the formation of films on the surface [1–4]. In this process, corrosion inhibitors take the role that a passivating oxide layer takes on several engineering materials, such as stainless steels or aluminum alloys [5]. Inhibitors may also actively participate in the electrode processes [1]. Corrosion inhibitors play an important role in mitigating corrosion, which causes huge economic losses [6]. The most potent corrosion inhibitors are based on carcinogenic Cr(VI) compounds, and environmentally friendly alternatives are needed [2,7].

Control experiments in a recent work on encapsulating poorly water soluble organic corrosion inhibitors in the cyclic oligosaccharide β-cyclodextrin (β-CD) for incorporation into model polymer coatings on zinc led to the surprising result that the coating delamination rate reduced also in the presence of pure β-CD [8]. This observation served as a motivation to investigate whether β-CD acts as corrosion inhibitor on its own. β-CD consists of seven α-D-1,4-linked glucosepyranose subunits, and is produced from starch by enzymatic conversion [9]. β-CD is not surface-active [10]. Although the use in food, medical, and pharmaceutical applications, including drug delivery, is well documented [11], application of CDs in corrosion protection is rare [8,12–14]. These applications mostly use CDs to solubilize hydrophobic corrosion inhibitors [8,12–13].

Metallic zinc is industrially used for cathodic protection of steel [15]. In this work, the inhibition of zinc corrosion by β-CD was investigated electrochemically. Inhibition efficiencies were determined by electrochemical impedance spectroscopy (EIS). After exposure to chloride containing electrolyte, samples were analysed by angle-dependent X-ray photoelectron spectroscopy (ADXPS) combined with ultraviolet photoelectron spectroscopy (UPS).

## Results and Discussion

**Figure 1:**
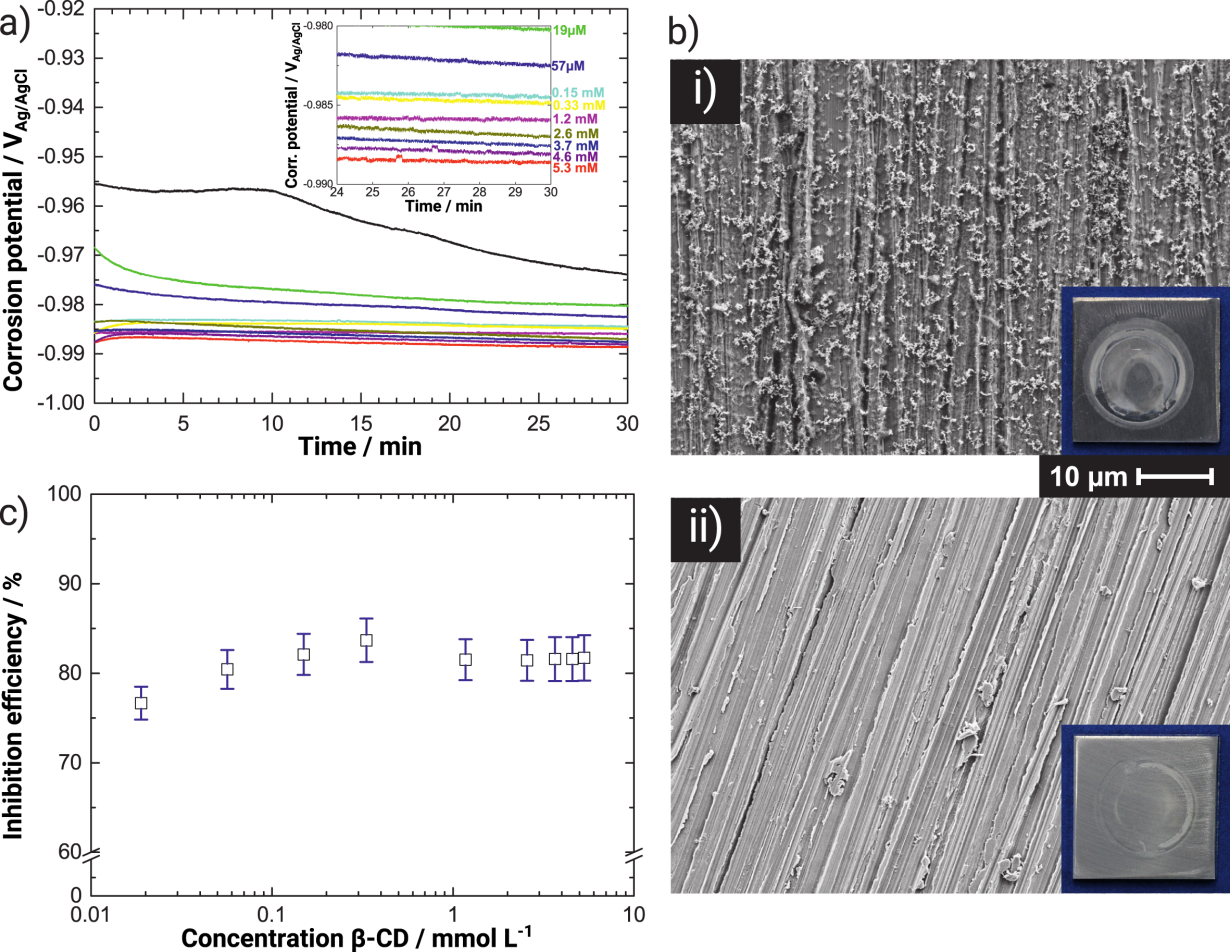
a) Evolution of corrosion potential *E*_corr_ with time *t* on zinc in aerated 0.1 M KCl with different concentration *c* of β-CD (concentration color coding in the inset). The inset shows a magnified version of the later stages of the experiments. The black line (top, excluded in the inset) is the reference experiment in the absence of β-CD. b) SEM images and optical micrographs (insets; sample width 2 cm) of the zinc surface after electrochemical experiments in 0.1 M KCl (i) and 0.1 M KCl + 5.3 mM β-CD (ii). Scale bars apply to both SEM images. c) Inhibition efficiencies η obtained from EIS data as a function of the β-CD concentration in 0.1 M KCl.

Electrochemical measurements of the corrosion potential *E*_corr_ displayed in Figure 1a show a cathodic shift by several tens of millivolts of the initial *E*_corr_ in the presence of β-CD in the electrolyte. Lower values of *E*_corr_ are an indication of a suppression of the cathodic process of oxygen reduction [16],

[1]
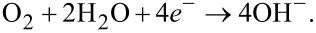


*E*_corr_ stablized quickly in the presence of the inhibitor, while reference measurements showed a slower decrease. Increasing the β-CD concentration decreased *E*_corr_ in the steady phase. Figure 1b (i) shows the state of the surface after exposure to 0.1 M KCl. In the presence of β-CD [Figure 1b (ii)], no precipitated corrosion products are visible. Comparison to the surface morphology before electrochemical experiments (Supporting Information File 1, Figure S1) shows that this morphology is retained after exposure to the electrolyte. The inhibition efficiencies η (Figure 1c), based on EIS data (Supporting Information File 1, Figure S2, Figure S3 and Table S1), show that with only 19 μM of β-CD in KCl, an inhibition efficiency of ≈75% is achieved. With an increase of β-CD concentration in the electrolyte, a further increase of η was observed. A maximum efficiency of ≈85% was found in the presence of 33 mM β-CD in the 0.1 M KCl solution. Overall, η ≈ 80% over a large concentration range.

**Figure 2:**
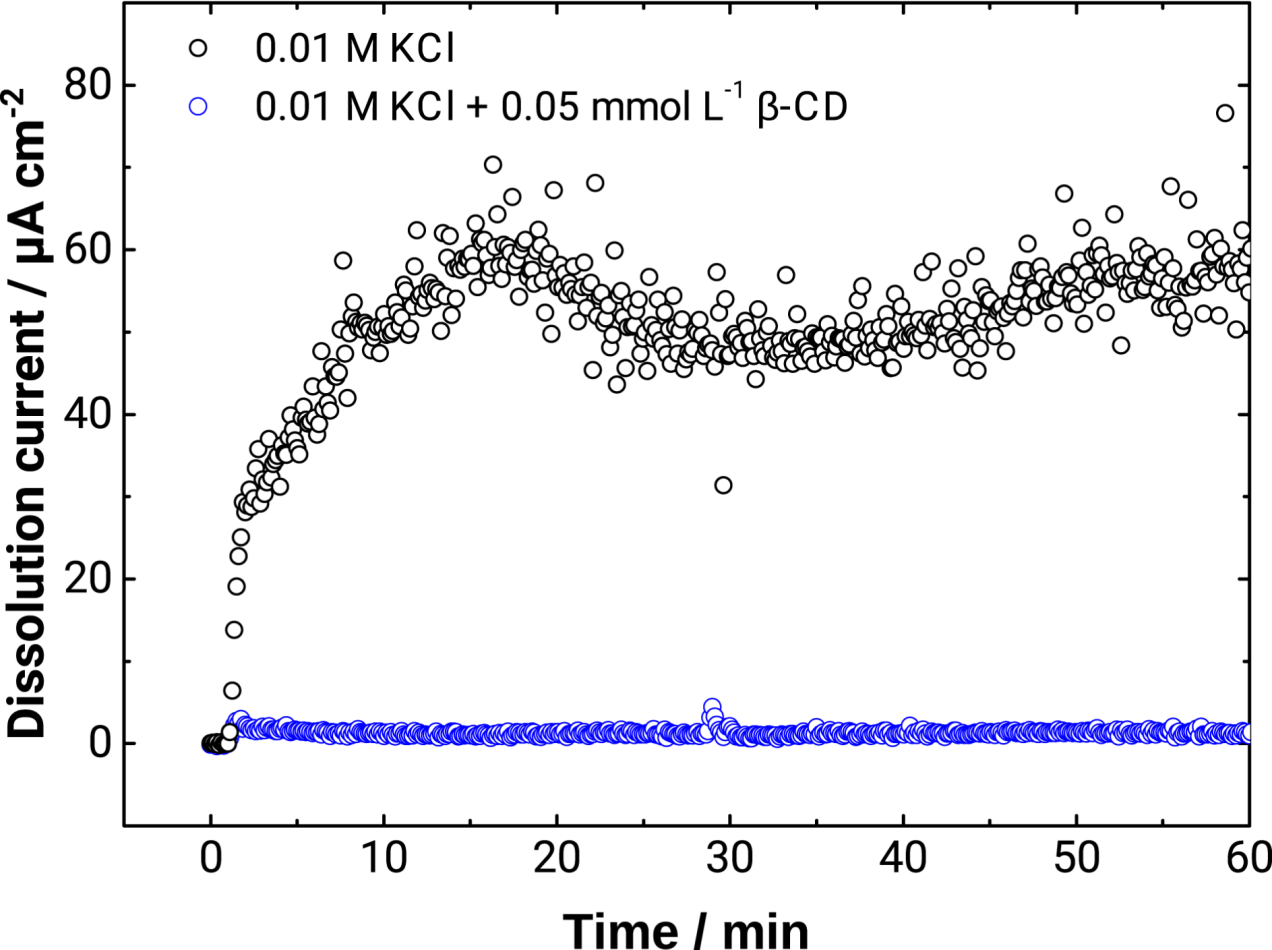
Dissolution current density *i*_diss_(Zn^2+^) of zinc measured by SFC/ICP-MS in 0.01 M KCl without β-CD and with 0.05 mM β-CD as a function of the time *t* during free corrosion experiments.

The impact of β-CD on the zinc dissolution was investigated using a scanning flow cell (SFC), with an inductively coupled plasma mass spectrometer (ICP-MS) downstream [17–18], which allows for the detection of dissolved species [19]. The KCl concentration was kept low, as high salt loads are challenging in experiments with ICP-MS online analytics. Figure 2 shows a significant decrease in the dissolution current of zinc in 0.01 M KCl, from 54 ± 5 μA·cm^−2^ in the absence of β-CD to 1.2 ± 0.3 μA·cm^−2^ in the presence of 0.05 mM β-CD. The zinc dissolution inhibition efficiency is thus ≈97% in 0.01 M KCl, where η *>* 90%. Consequently, the inhibition of the oxygen reduction and the concomitant shift in *E*_corr_ leads to a significant decrease of the anodic dissolution,

[2]
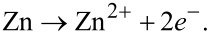


β-CD thus acts as a mixed corrosion inhibitor. It must be stressed that due to the difference in conditions –stagnant vs flowing electrolyte, chloride concentration– compared to the EIS measurements a quantitative comparison of the efficiencies is not reasonable.

In situ spectroscopic ellipsometry experiments conducted both in 0.1 M KCl as well as in 0.1 M KCl with 5.3 mM β-CD show no formation of an adsorbate layer on the samples (Supporting Information File 1, Figure S6). On the other hand, ex situ Raman spectra (Supporting Information File 1, Figure S5) recorded after exposure do show the presence of β-CD on the surface, by the presence of several of the characteristic vibrational modes in the spectrum.

The dissolution product Zn^2+^ is a reactant in follow-up chemical reactions, forming precipitates such as hydrated zinc oxide [15]. ZnO is naturally an n-type semiconductor with a band gap of 3.4 eV [20]. Oxides formed in an aerated corrosion process are typically defect-rich oxides [21], especially in the presence of Cl^−^ [15]. Consequently, the products remain initially conductive, not inhibiting further corrosion. The oxide formed on metallic Zn has noticeably different properties than crystalline bulk ZnO, due to the presence of different point defects, which have a strong effect on the electronic structure of the oxide [21–22].

**Figure 3:**
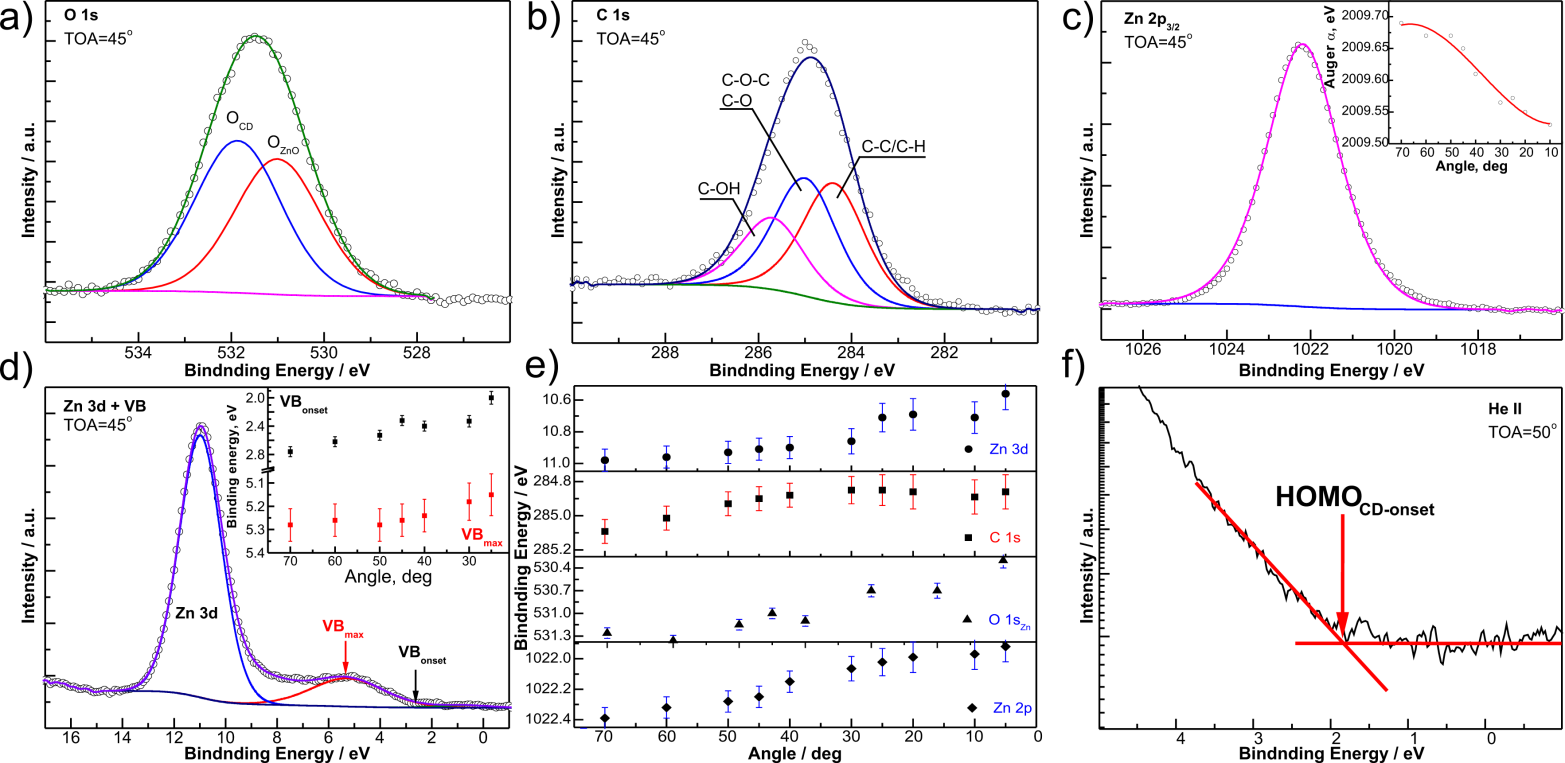
High resolution XP spectra at TOA = 45° of β-CD/ZnO/Zn after 24 h of exposure to 0.1 M KCl + 5.3 mM β-CD; (a) O 1s energy region, (b) C 1s region, (c) Zn 2p*_3/2_*, (d) Zn 3d - valence band (VB) energy region, with ZnO VB onset and ZnO VB maximum as functions of the TOA (inset). (e) Binding-energy variations for recorded spectral regions with TOA; (f) UPS HOMO onset of β-CD recorded with He II excitation. The Auger parameter α is shown as inset in (c).

ADXPS was utilized to understand defect levels, electronic structure, and chemical composition of the zinc surface, based on a previously established method [23–24]. Results from the β-CD/ZnO system are shown in Figure 3. Take-off-angles (TOA) close to 90° probe deeper into the volume of the sample, while low TOAs weigh surface contributions stronger. Although there are procedures that allow for a quantitative analysis of the depth dependence of photoemission information [23,25], material constants that are not exactly known for the β-CD/ZnO system are required for application of these procedures. Therefore, the presented depth information is an estimate, based on typical parameters for organic compounds (see Experimental section). Space-charge effects can be probed by ADXPS [26–27], in which the signal is dependent on many source- and sample-specific parameters. Among others, the most important are the number of electrons per pulse, the spot size on the sample, the pulse duration, the initial energy, and the angular distribution of the photoelectrons [26–27]. The difficulties in space-charge layer probing by ADXPS are related mainly to angular broadening of the particular components while measuring with varying TOA [26–27]. For quantification, we assumed isotropic emission of the cloud electrons and surface-normal emission of the test electrons, i.e., for the majority of the photoemission signal. The angular distribution is assumed to be equally simple [26]. The rest of the parasite effects are expected to be rather low for the probed electron densities [27].

The qualitative analysis of the O 1s region (Figure 3a) shows two main components, which can be attributed to oxygen from ZnO and β-CD. The relative intensities of the two different components at different TOA (Supporting Information File 1, Figure S4a) is consistent with the expectation that β-CD is on top of ZnO: At low TOA, only a small peak from ZnO is detected, while at larger TOA, the ZnO fraction dominates the signal. ZnO domination also proves the β-CD layer to be thinner than the oxide layer. The C 1s peak (Figure 3b) can be decomposed into three main components assigned to C–C/C–H, C–O–C/C–O and C–OH bonds [28], as expected for the chemical composition of β-CD. The latter two components are different from what is typically observed as adventitious carbon in XPS measurements after transfer through ambient air. Therefore, the resulting signal does not originate mainly from impurities collected through the sample transfer. Due to the high symmetry of the Zn 2p*_3/2_* peak, analysis of the Auger parameter α was needed to understand the electronic structure of the layer (Figure 3c). Figure 3d shows the Zn 3d region, including an inset with the depth dependence of the ZnO valence band (VB) edge region. The ZnO VB region contains contributions from overlapping Zn 4s/4p, O 2p and C 2p levels [29–30]. Because of (i) the significantly bigger excitation cross-section for ZnO compared to β-CD, and (ii) the expected atomic contributions to the VB region, this region can be treated as originating mainly from ZnO [31]. The decomposition of this region for different TOA is shown in Figure S4b (Supporting Information File 1).

The binding energy of all examined energy regions (Figure 3d inset and Figure 3e) shows the shifts as a function of the TOA. Depth-dependent shifts are consequently present for the main core levels attributed to ZnO (Zn 3d, Zn 2p) and β-CD (C 1s). A common tendency is a shift of the levels towards the lower binding energies with decreasing depth. A similar trend was observed for the VB levels. From TOA = 70° to 45°, the onset of the VB shifted by ≈0.5 eV, while the peak energy VB_max_ remained unchanged. The latter shows a slight but systematic decrease by ≈0.1 eV towards lower TOA. Our former experimental studies [32], as well as theoretical analysis [33], shows that such a situation is encountered when defect levels contribute to the XP spectra. For higher TOA probing deeper into the film, the defect contribution is suppressed by the dominating rest of the photoemission signal, while for low TOA probing surface regions, the defects are dominating. While the variation of binding energies comprises both chemical shift and changes in the local electrostatic potential [34], the evident difference in the shift slope for the different examined signals (e.g., ZnO and β-CD related components of O 1s, Figure S4c, Supporting Information File 1) shows that charging effects can be ruled out. Charging can also be ruled out as it should cause the same energy shift for all energy regions [35], as opposed to what is observed.

Since the XPS was not sensitive for the region of the highest occupied molecular orbital (HOMO) of β-CD, this region was analyzed by UPS. Figure 3f presents the magnification of the low-energy onset of the UP spectra, which allows for determining the energetic difference between β-CD HOMO and Fermi level *E*_F_. The facts that β-CD is present on the top surface, and that UPS probes only the surface region make it a reasonable assumption that β-CD dominates the spectra in this region, based on a molecular diameter of ca. 1 nm.

**Figure 4:**
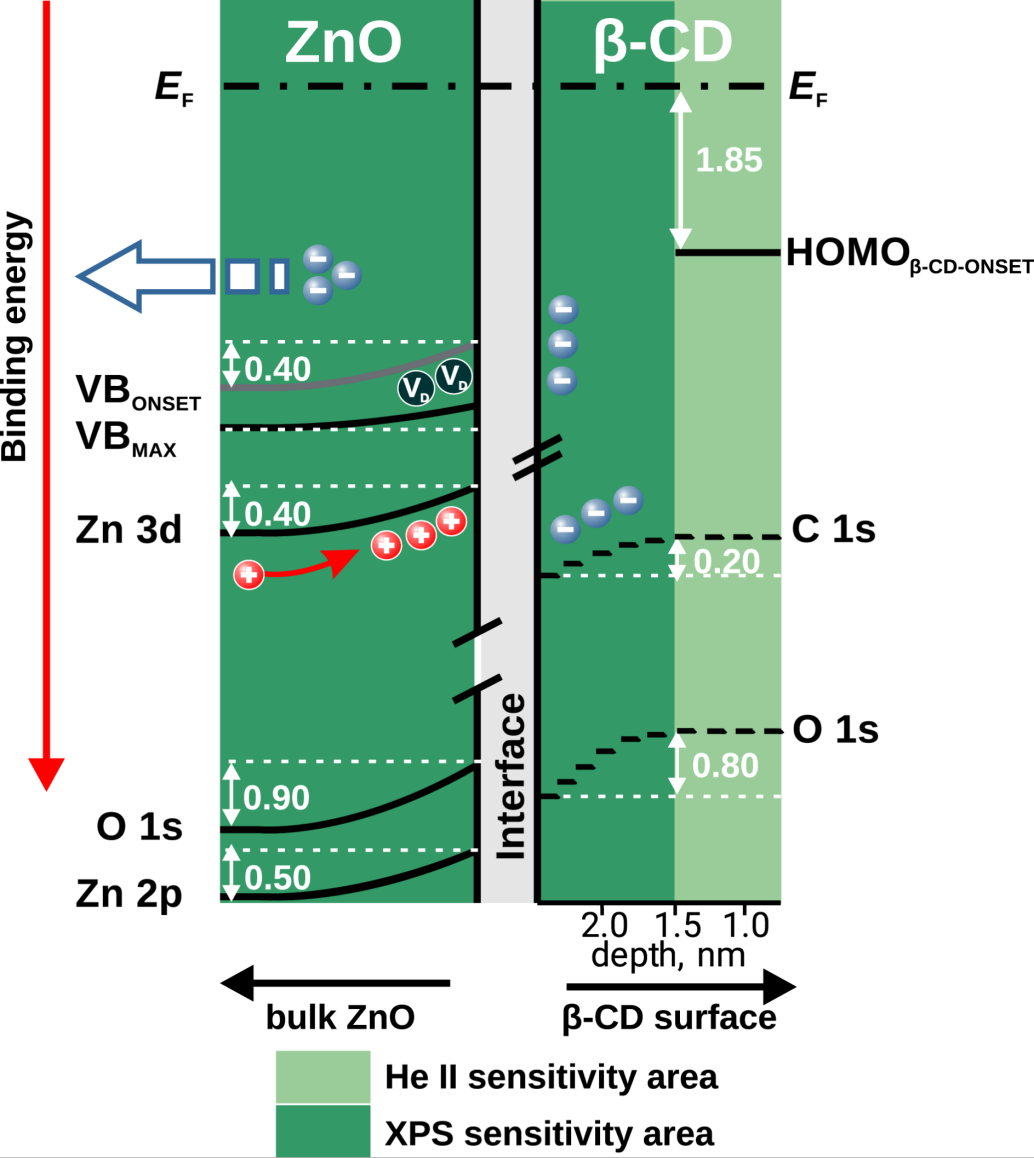
Band diagram of the β-CD/ZnO interface constructed from photoemission experiments, with energy levels in electronvolts. XPS and He II UPS sensitivity areas are distinguished by the different color intensity, the given thickness values are rough estimates only. The upward energy level shift in ZnO is a consequence of positive charge accumulation at the interface. On the β-CD side, negative charge accumulation causes a downward energy level change.

With the assumption of a common *E*_F_ of sample and electron energy analyzer, the photoemission data were used to construct the diagram showing the depth-dependent changes in the electronic structure of the sample (Figure 4). Two main probing depths were distinguished, the XPS sensitivity area at medium TOA, and the He II UPS sensitivity area. Band bending was observed at the interface between the ZnO thin film and β-CD on top. While the Zn 3d and VB energy changes are ≈0.40 eV, Zn 2p as the main Zn core level shifts ≈0.50 eV towards lower binding energy. The different traces of VB_onset_ and VB_max_ are attributed to an effect of the defect levels. The most probable defects are zinc vacancies V_Zn_, as these have the lowest formation energy among the native point defects in ZnO [36–37]. V_Zn_ can be treated as deep acceptors, hence they should be manifested close to the top of the VB of ZnO [36–37]. A possible alternative explanation is a contribution from the β-CD HOMO to ZnO VB_onset_ changes, since the β-CD HOMO onset was found 1.85 eV from the Fermi level. Intuitively, one would assume oxygen vacancies or zinc interstitials as dominating defects in the predominately n-type ZnO.

The largest energy shift was observed in the O 1s region for both components. The ZnO related components shift by ≈0.90 eV, almost twice as much as those of the Zn 2p and 3d levels. The β-CD related C 1s level shift is only 0.20 eV. Energy level shift is a sign of charge dislocation across the β-CD/ZnO interface. (As explained above, defect levels affect the VB_onset_ and VB_max_ energy positions differently, consequently altering the density of states in the VB region. Altered density of states leads to different charge carrier densities at the interface and a decrease in potential barrier for charge dislocation.) From the magnitude of the energy level shift, oxygen must be the main center for the charge dislocation. It is highly probable that charge is drained from the n-type ZnO layer towards the β-CD layer, which consequently behaves like a p-type system in this situation. This conclusion is supported by He II UPS, showing *E*_F_ − HOMO-CD_onset_ to be 1.85 eV. The band gap of β-CD has been reported to be 12.44 eV [38], and judging from the position of the Fermi level, β-CD is in the same role as a p-type system. Charge dislocation must lead to hole accumulation at the ZnO side, i.e., depletion of negative charge. On the β-CD side, negative charge must be accumulated, either by electron accumulation or by molecular polarization. The latter is more likely, as the amount of free charge carriers in a molecular insulator such as β-CD is expected to be rather low. As the O 1s peak of β-CD is most affected by the charge dislocation, it is likely that β-CD accepts the charge via the hydroxy groups. The charge dislocation explains the observed energy level shift in photoemission experiments, closely resembling band bending. From the derived band diagram, charge dislocation could also take place via holes. Due the lower mobility of holes, and the nature of ZnO, however, electrons are more likely to be the dominating species. In the case of a thick oxide layer, generation of a large polarization at the interface is a further possibility. No evidence exists here for such a polarization, and it is unlikely for the investigated case with a thin oxide. A band bending of ca. 0.1 eV is usually observed in oxides at interfaces and also in air [39]. During corrosion processes, electrode polarization lifts the band bending to the active corrosion potential [39].

The consequence of the energy level bending makes the ZnO less an n-type semiconductor than the defect-dominated thin film initially is, and transforms it towards an intrinsic semiconductor. Decrease in defect concentration decreases also charge carrier concentration, and consequently the oxide conductivity. This transformation hinders a continuous current flow across the interface, which would be needed in a corrosion process. As a consequence, β-CD inhibits zinc corrosion by modifying the oxide. Lower conductivity of the oxide should lead to lower current densities during corrosion processes. Electrochemically speaking, the inhibition is most likely realised by a decrease in the apparent exchange current densities of cathodic and anodic partial reactions as a result of the modified oxide defect density. Furthermore, lower defect density makes vacancy coalescence as the first step of breakdown of an oxide, e.g., in pitting corrosion, less likely.

## Conclusion

β-CD shows inhibition efficiencies of up to 85% against corrosion of zinc in neutral KCl. The inhibition mechanism is based on a complex interfacial process. β-CD adsorbed to the oxide-covered metal, resulting in a static charge dislocation across the β-CD/ZnO interface. This charge dislocation causes energy level shifts near the interface, making the interface behave similarly to an n–p junction. Charge transport is hence only possible from the n-type ZnO to β-CD, which behaves like a p-type layer, effectively blocking the anodic reaction. Most decisively, the energy level shift induced by the changes in the defect chemistry because of β-CD adsorption is too high to enable easy electron transfer at active corrosion conditions. This work shows that the energy level alignment across the interface can be significantly affected by the presence of simple organic molecules. The defect chemistry of the oxide plays also an important role in this context. This mechanism of corrosion inhibition was previously undescribed and may be exploited systematically in design of inhibitors.

## Experimental

Zinc sheets (99.95%; Goodfellow; thickness 1.5 mm) were cut to a size of 2 cm × 2 cm, ground with 1000P SiC paper (1000P), cleaned with soap water and ethanol, finally dried under a nitrogen stream, and freshly used for electrochemical experiments. β-CD and KCl were purchased from Sigma-Aldrich and used as received. Electrolytes were prepared using ultrapure water (USF ELGA; conductivity below 0.055 μS·cm^−1^) to the final concentrations of KCl and β-CD. All experiments were done at ambient temperature of 23 ± 2 °C.

Electrochemical measurements were conducted in a classical three-electrode setup, with stagnant electrolyte saturated with air before the experiment, using a commercial Ag/AgCl/3 M KCl reference electrode (Metrohm) in a Luggin capillary, a platinum foil counter electrode, and a zinc working electrode with an exposed surface area of 0.196 cm^2^. The electrolyte volume for the measurements was 10 mL, and the electrolyte surface was ≈2.5 cm away from the surface of the working electrode. All electrode potentials shown here are referenced against Ag/AgCl/3 M KCl. Experiments were conducted using a Solatron multichannel potentiostat system (Solatron 1255B Frequency Response Analyser, Solatron Muliplexer 1281, and Solatron 1286 Electrochemical Interface). EIS was executed with an AC amplitude of 10 mV vs open-circuit potential in a frequency range from 10^4^ to 10^−1^ Hz. The impedance spectra were fitted using the software ZView. The resulting impedance spectra and the equivalent circuit for data fitting are shown in Figure S2 (Supporting Information File 1). From EIS data, the corrosion current densities *i*_corr_ were estimated as given on page 48 of [16] as

[3]
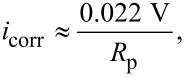


from the area-normalized polarization resistance *R*_p_. The latter was determined from fitting the EIS data. The inhibition efficiency η was calculated as

[4]
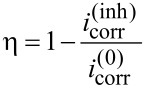


from the corrosion current densities 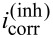 and 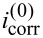 recorded in the presence and absence of the inhibitor β-CD, respectively. The derivation of Equation 3 assumes equal cathodic and anodic Tafel slopes of 0.1 V per decade. Corrosion current densities determined according to Equation 3 deviate consequently “typically not more than a factor of 2 from those determined using the real Tafel slopes” [16] (p. 48). Using the simplified Equation 3 circumvents the need to determine a Tafel slope for zinc dissolution. Importantly, the trend within a series of experiments will be correctly reproduced if the dissolution mechanism and hence the Tafel slope remains the same, as is expected for this system.

The dissolution of zinc was monitored by SFC coupled to an ICP-MS. SFC/ICP-MS is a setup which allows for simultaneous electrochemical measurements and analysis of the dissolved amount of metal. This setup was used for various applications including corrosion characteristics of metals in different electrolytes [19,40–41]. In the scope of this work, the SFC/ICP-MS was used to determine the dissolution of zinc in the presence and absence of β-CD in a free corrosion process, i.e., at open circuit. The concentration *c*_Zn2+_ of dissolved zinc species was detected by a mass spectrometer. The dissolution current density *i*_diss_(Zn^2+^) was calculated as

[5]
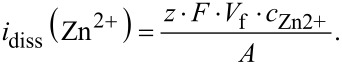


Here, *z* is the charge transfer number (for Zn, *z* = 2), *F* the Faraday constant, *V*_f_ is the volume flow rate and *A* the area of the working electrode. The experiments were conducted using the cell design described elsewhere [17–18]. The cell was equipped in a classical manner: A platinum wire was set as a counter electrode, a commercial Ag/AgCl/3 M KCl electrode was placed as reference electrode, and zinc foil as the working electrode. The area *A* of the working electrode exposed to the electrolyte was 0.21 mm^2^. The experiments were performed in 0.01 M KCl solution at a flow rate *V*_f_ = 166 μL·min^−1^. As hardware an ICP-MS system (XexION 300X, PerkinElmer) and a Gamry Reference 600 potentiostat were employed. The ICP-MS data were recorded for ^64^Zn and ^68^Zn with ^74^Ge as internal standard. The dissolution inhibition efficiency was calculated as defined in Equation 4 after replacing *i*_corr_ by *i*_diss_(Zn^2+^).

SEM inspections of the zinc surface after electrochemical testing were carried out with a Zeiss LEO 1550 VP at an acceleration voltage of 10 kV and at a working distance of 5–7 mm. Samples for SEM were Zn sheets after EIS measurements in an electrolyte containing 0.1 M KCl with and without 5.3 mM β-CD. The samples were left exposed to the solution after the EIS measurement finished, such that they were exposed to the solution for 24 h in total. The samples were rinsed afterwards with excessive amounts of deionised water from a wash bottle. Care has been taken to rinse the different parts of the surfaces evenly, and such that the samples were overall rinsed for at least 30 s. After rinsing with water, the surface was briefly rinsed with absolute ethanol to dissolve loosely bound organic compounds. Subsequently, the samples were dried first in a nitrogen stream and later in a desiccator in vacuum.

ADXPS was carried out on a Physical Electronics PHI Quantera II spectrometer equipped with an Al Kα micro-focused source at 1486.74 eV. Samples for ADXPS were prepared in the same manner as for SEM. In ADXPS, to compensate X-ray source-induced charging effects, a dual-beam charge neutralizer was applied. The pass energy was set to 140 eV for the survey spectra, with a step of 0.4 eV, and to 26 eV for individual energy regions, with an energy step 0.05 eV. The XPS system base pressure was 2·10^−8^ Pa. All XPS spectra were recorded with take-off angles (TOA; defined as the angle between analyzer axis and the sample plane) varying from 70° to 5°. For XPS experiments, the approximated photoelectron attenuation length λ_a_ for organic substances in the photoelectron kinetic energy range of 500–1500 eV is assumed to be ≈2.8 nm [42], hence the information depth 3λ_a_ is ≈8.4 nm for TOA ≈ 90°. As the information depth 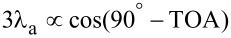 [25], at high TOA the experiment probes deeper into the layer, while experiments at low TOA are more sensitive to the surface. XPS data were fitted using CASA XPS software. Each peak was represented by a sum of Gaussian (70%) and Lorentzian (30%) lines. The secondary electron background was subtracted with the Shirley function. The probing depth estimation was performed with the following assumptions: (i) About 65% of the emitted X-ray- or UV-excited electrons originate from a depth of less than λ_a_ [43], (ii) diffraction and scattering effects for the photoelectron transfer towards the sample surface are negligible, (iii) both oxide and organic layer are continuous, (iv) the X-ray flux intensity is not attenuated significantly throughout the analyzed layers, and (v) λ_a_ for particular photoelectrons are treated as constants within the examined films.

Deeper insight into the interfacial energy levels of oxidized Zn was obtained from comparing the kinetic energy *E*_kin_(*jkl*) of the main Auger transition *jkl*, here Zn LMM, with the binding energy *E*_B_(*i*) of a photoelectron from the main atomic level *i*, here Zn 2p [44–45] as

[6]
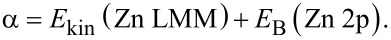


The Auger parameter α is a measure in the differences in Pauling electronegativity [46]. Briefly, the lower α, the higher is the oxidation state of the species [44–45].

UPS measurements were done for probing only the outer surface of the system, utilizing the He II spectral line at 40.8 eV of an UV source (SPECS UVS 300). The emitted photoelectrons were detected with a hemispherical electron energy analyzer (SPECS PHOIBOS 150) with TOA = 50°. This configuration gives an information depth of ≈1.5 nm [43].

Raman spectroscopy was performed on samples prepared in the same manner as for SEM. Spectra were recorded using a Witec alpha300M confocal Raman microscope. The samples were irradiated with an excitation wavelength of 532.1 nm/2.33 eV through a microscope objective of 100× magnification and with a numerical aperture of 0.75.

In situ spectroscopic ellipsometry (SE) was performed on Zn samples polished down to a 1 μm diamond suspension. Experiments were performed at an angle of incidence of 70° using a Sentech Instruments SE 800 spectroscopic ellipsometer working in the wavelength range of 280–810 nm (1.5–4.4 eV). The details of the used in situ cell were described elsewhere [47–48]. Experiments were done in 0.1 M KCl, both in the presence and absence of 5.3 mM β-CD, under open-circuit conditions. The electrolyte was externally purged with argon, and pumped through the cell with a rate of 2 mL·min^−1^ using a peristaltic pump (Ismatec IDEX Health and Science). During the measurement, the pump rate was reduced to 10.6 μL·min^−1^. In this work, the duration of a single ellipsometric measurement was ca. 25 s. Several analysis methods have been tested on the resulting data, as described previously [48–50]. In total, three repetitions of the experiments have been carried out.

## Supporting Information

File 1 Additional experimental data.
Supporting information shows the control SEM image, detailed results of EIS measurements, detailed peak decomposition of ADXPS data, Raman spectra, and in situ spectroscopic ellipsometry data.
