# Supplementary material for: Cyclodextrin inhibits zinc corrosion by destabilizing point defect formation in the oxide layer
Source: Beilstein J Nanotechnol. 2018 Mar 20;9:936–44. doi: 10.3762/bjnano.9.86 (PMC5870151; doi:10.3762/bjnano.9.86)
Supplement: File 1 — Additional experimental data. Supporting information shows the control SEM image, detailed results of EIS measurements, detailed peak decomposition of ADXPS data, Raman spectra, and in situ spectroscopic ellipsometry data. [file Beilstein_J_Nanotechnol-09-936-s001.pdf]

Supporting Information

for

Cyclodextrin inhibits zinc corrosion by destabilizing point defect formation in the oxide layer

Abdulrahman Altin[1], Maciej Krzywiecki[1,2], Adnan Sarfraz[1], Cigdem Toparli[1], Claudius Laska[1], Philipp Kerger[1], Aleksandar Zeradjanin[1,3], Karl J.J. Mayrhofer[1,3], Michael Rohwerder[1] and Andreas Erbe*[1,4]

Address: [1]Max-Planck-Institut für Eisenforschung GmbH, Max-Planck-Str. 1, 40237 Düsseldorf, Germany; [2]Institute of Physics–CSE, Silesian University of Technology, Konarskiego 22B, 44-100 Gliwice, Poland; [3]Forschungszentrum Jülich GmbH, Helmholtz Institute Erlangen-Nürnberg for Renewable Energy (IEK-11), Egerlandstraße 3, 91058 Erlangen, Germany and [4]Department of Materials Science and Engineering, NTNU, Norwegian University of Science and Technology, 7491 Trondheim, Norway

Email: Andreas Erbe - CD-inhibitor-mechanism@the-passivists.org

* Corresponding author

Additional experimental data

SEM reference image

The surface of a zinc sample prepared for the corrosion experiments is shown in Figure S1. The image shows the typical patterns obtained after grinding the sample. The pattern is still visible on the sample exposed in the presence of β-CD shown in Figure 1b of the main manuscript.

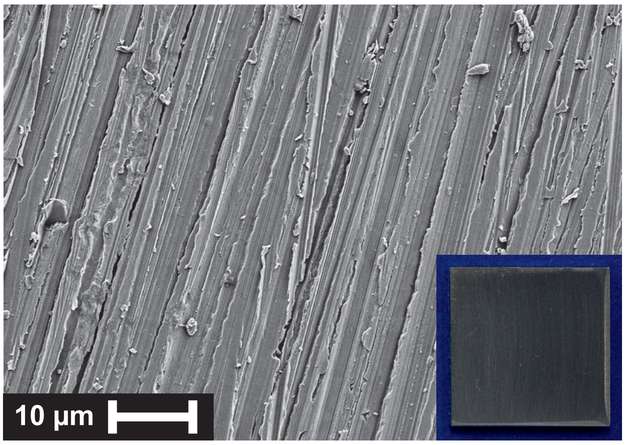

Figure S1: SEM image and optical micrograph (inset) of a zinc surface as prepared for the corrosion experiments. This sample has not been exposed to any electrolyte before.

Electrochemical results

A compilation of EIS results of zinc samples in 0.1 M KCl in the presence of different concentrations of β-CD is shown in Figure S2, together with the equivalent circuit used for fitting the data. The results of the fits are compiled in Table S1. The parameters obtained are solution resistance R_s, polarization resistance $R_p{}^*$, and Q_0 and γ of the constant phase element (CPE) with impedance $Z = 1/[Q_0(I\omega)^\gamma]$, where $I = \sqrt{-1}$. $R_p{}^*$ was subsequently normalized to the electrode area A as $R_p = R_p{}^* \cdot A$ and used to calculate the corrosion current density according to Equation 3. Both R_p and i_{corr} as a function of added β-CD concentration are shown in Figure S3.

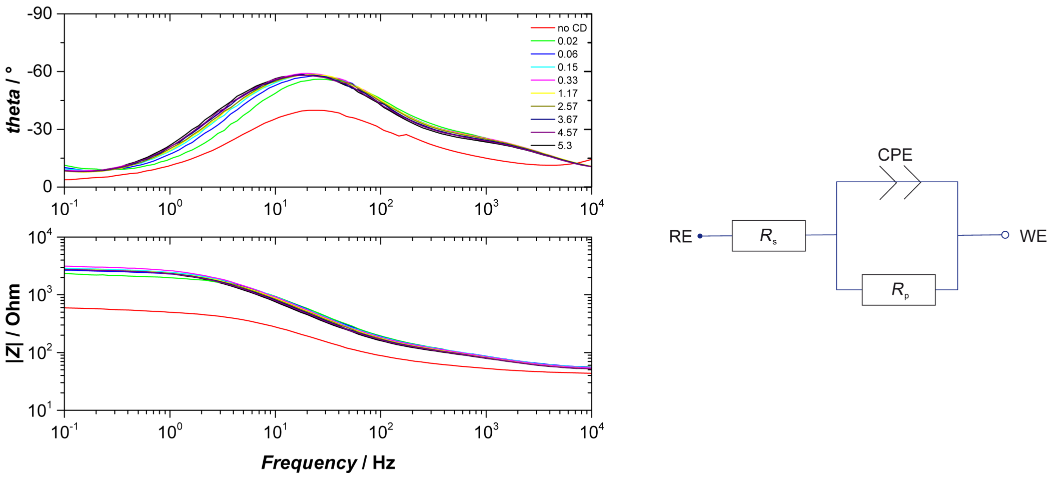

Figure S2: (left) The Bode plots recorded on zinc in the presence of different concentrations of β-cyclodextrin in 0.1 M KCl and (right) the equivalent circuit used for the fitting of the impedance spectra. The numbers in the graph indicate the concentration c of β-CD, in mmol L^{-1}.

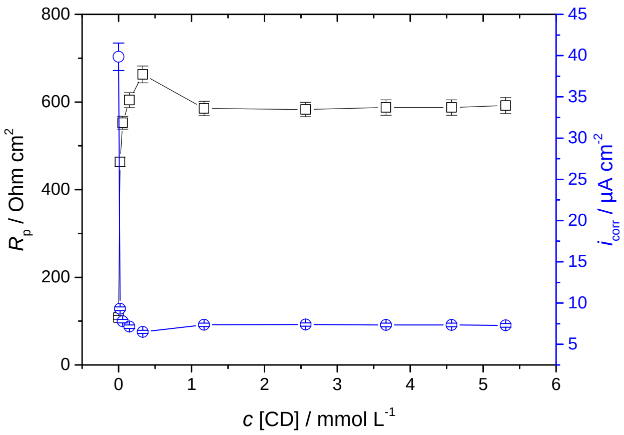

Figure S3: Polarization resistance R_p and corrosion current density i_{corr} on zinc in 0.1 M KCl as function of the concentrations of added β-CD.

Table S1: Obtained parameters and respective standard deviations δX, where X stands for the respective quantity, obtained from fitting the EIS data shown in Figure S2 for different concentrations $c_{\beta\text{-CD}}$ of CD, using the equivalent circuit shown also in Figure S2. Parameters are defined in the equivalent circuit

c_{CD} mmol L^{-1}	R_s Ω	δR_s Ω	$R_p{}^*$ Ω	$\delta R_p{}^*$ Ω	Q_0 $As^{\gamma}\,V^{-1}$	δQ_0 $As^{\gamma}\,V^{-1}$	γ	$\delta\gamma$
0	50	2	550	20	2.1E-04	2E-05	0.67	0.02
0.02	54	1	2360	60	4.7E-05	2E-06	0.73	0.01
0.06	55	2	2820	80	4.8E-05	2E-06	0.73	0.01
0.15	54	1	3080	90	4.9E-05	2E-06	0.73	0.01
0.33	54	1	3380	100	5.0E-05	2E-06	0.73	0.01
1.17	52	1	2990	80	5.0E-05	2E-06	0.73	0.01
2.57	52	1	2970	90	5.4E-05	3E-06	0.73	0.01
3.67	52	1	3000	90	5.6E-05	3E-06	0.73	0.01
4.57	52	1	3000	90	5.8E-05	3E-06	0.73	0.01
5.31	52	1	3020	90	6.0E-05	3E-06	0.73	0.01

ADXPS results

Examples of XP spectra obtained at different take off angles (30°, 45° and 70°) are shown in Figure S4 for the O 1s region and the region including the Zn 3d and valence band (VB) contributions. The O 1s region can be decomposed into two components. The higher binding energy component corresponds to organic oxygen, while the lower binding energy component to the oxide oxygen. The maximum of the respective components shift with take off angle (Figure S4c), hence is different in different depths of the sample. The decomposition of the VB region shows a significant shift with take off angle of the onset of the ZnO VB (VB_{onset}), and a moderate shift of the maximum of the ZnO VB (VB_{max}). The shifts with TOA have been used to construct a depth-dependent band-like diagram, which is presented in the main manuscript.

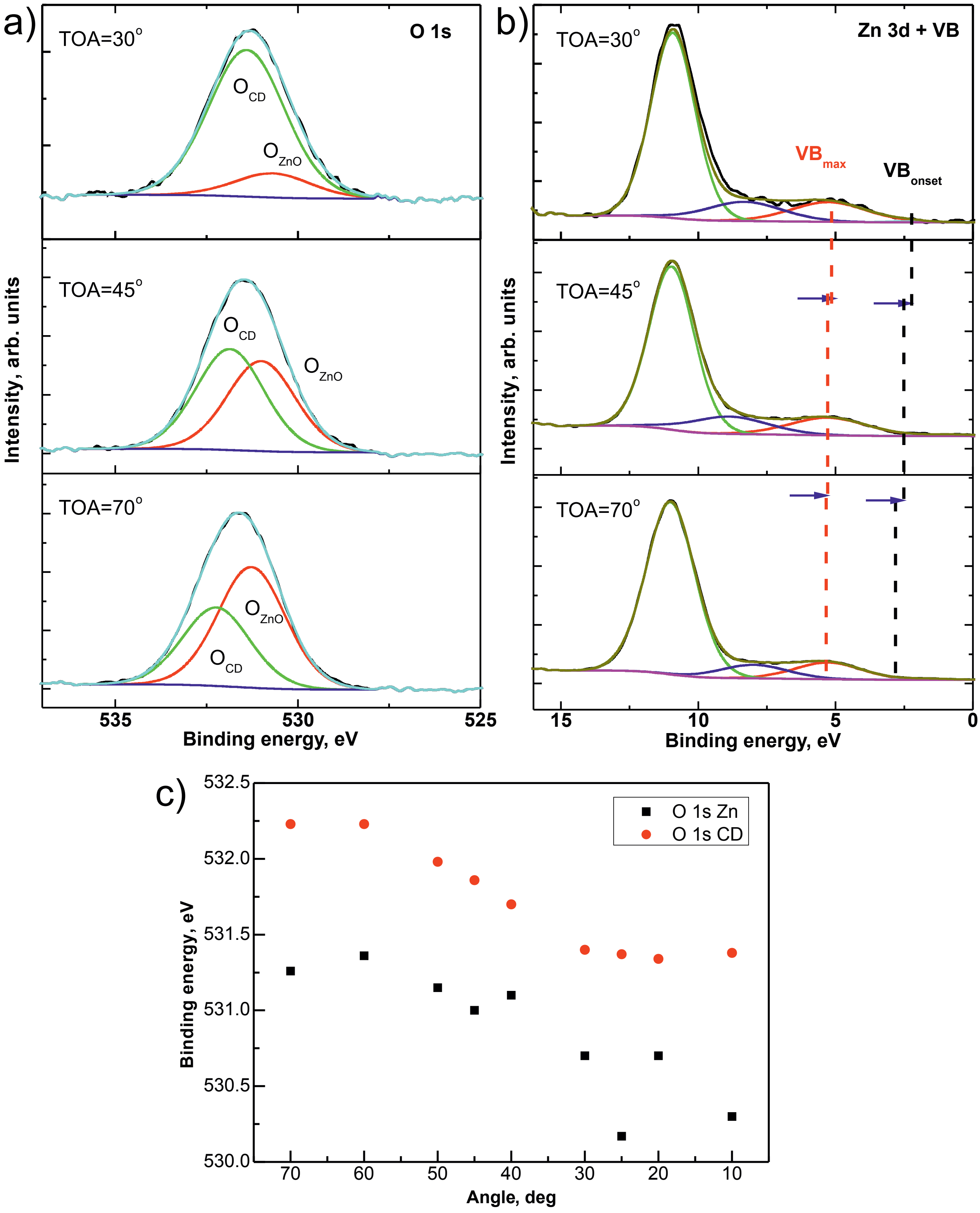

Figure S4: ADXPS for β-CD/ZnO/Zn. (a) Decomposition of the O 1s binding energy region for three TOA. (b) Decomposition of the Zn 3d-VB region for three TOA. (c) Plot of the binding energy for the two O 1s components as function of the TOA.

Raman spectra

A Raman spectrum of a zinc surface after exposure to 0.1 M KCl with 5 mM β-CD is shown in Figure S5. The spectra show the essential features present in the β-CD powder, and features related to ZnO. Naturally, β-CD features are absent in the control experiment, in which a sample was exposed to 0.1M KCl in the absence of β-CD. Consequently, β-CD is present on the Zn surface after exposure to a solution containing β-CD. A detailed interpretation of the spectra is beyond the scope of this work.

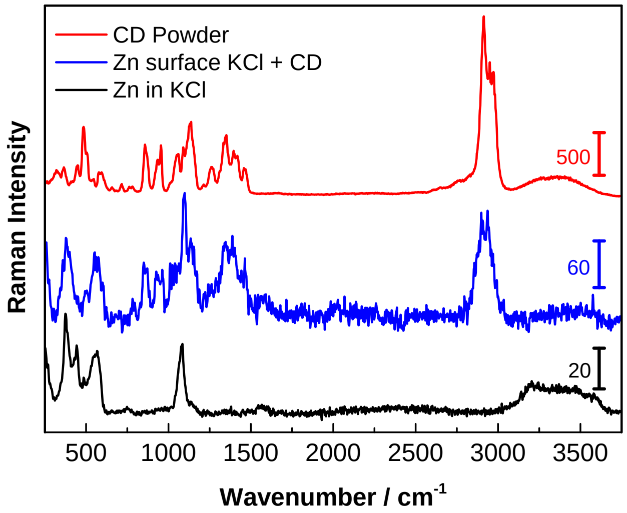

Figure S5: Raman spectra of β-CD powder, Zn after exposure to 0.1 M KCl and Zn after exposure to 0.1 M KCl with 5 mM β-CD, as indicated in the graph. Spectra have been vertically offset for clarity.

In situ ellipsometry results

Figure S6a shows the raw data of the ellipsometric parameter Δ, averaged over a wavelength range of 690–710 nm, as a function of exposure time to 0.1 M KCl containing 5.3 mM β-CD. The averaging was performed to reduce the effect of spectral noise. The wavelength at the upper end of the visible range was chosen as all involved species except metallic zinc are non-absorbing in this wavelength range. Despite the averaging performed, the data remains rather noisy. This kind of noise is typically not observed in measurements of zinc or similar systems performed on the same setup, both ex situ [1,2] and in situ [2-8]. We attribute the observed noise to the presence of ongoing dissolution and ref-

ormation of the surface layer on zinc, e.g. as in pitting corrosion. A growing oxide layer on a metal leads mostly to a parallel shift in Δ [3,4,9], i.e., the shift in Δ is only weakly wavelength-dependent. Consequently, it is in first approximation justified to average Δ over the complete measured wavelength range (Figure S6b). In this way, effects on the spectra of the change of optical constants of the film with wavelength are neglected. Differences in the initial Δ may be related to differences in roughness in the surface preparation chosen here [10].

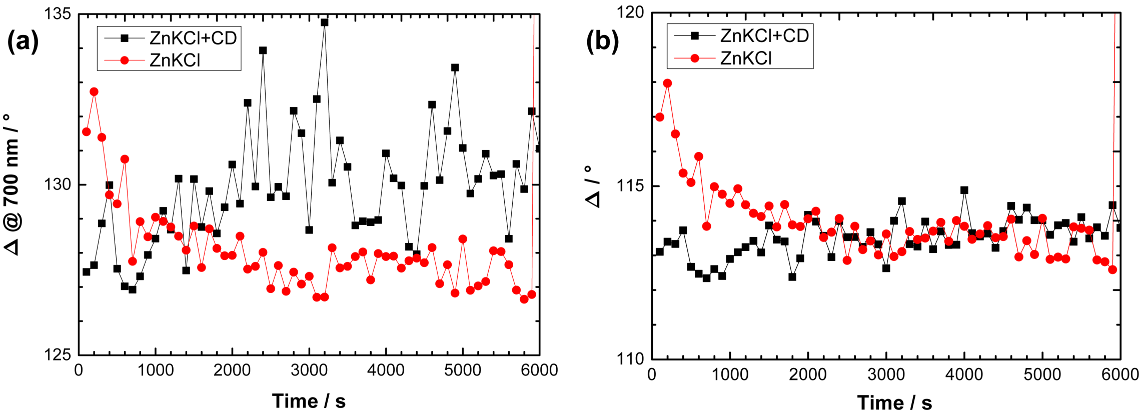

Figure S6: Ellipsometric parameter Δ as function of time for zinc exposed to 0.1 M KCl (red circles) and 0.1 M KCl containing 5.3 mM β-CD (black squares). (a) Δ averaged over a wavelength range of 690–710 nm, (b) Δ averaged over the complete measured wavelength range of 300–820 nm.

Simulations were performed of expected ellipsometric spectra for the system water–ZnO–Zn, in analogy to the calibration approach in data analysis used previously to determine the thickness of copper oxides on copper [5]. Optical constants of water [11], ZnO [12], and Zn [1] were all used from published resources. As a result, the parameter Δ at a wavelength of 700 nm was plotted as function of oxide thickness (Figure S7). Δ changes by approx. -1.5 °/nm. The curve of Δ for Zn in pure KCl corresponds to a growth of an oxide of ≈8 nm over the experiment. In the presence of β-CD, no clear signature of growth is observed. This absence of corrosion product formation can be attributed to the inhibition of oxide growth by β-CD. On the other hand, there is no indication that this corrosion product growth is replaced by a stable film of β-CD on the surface beyond monolayer coverage.

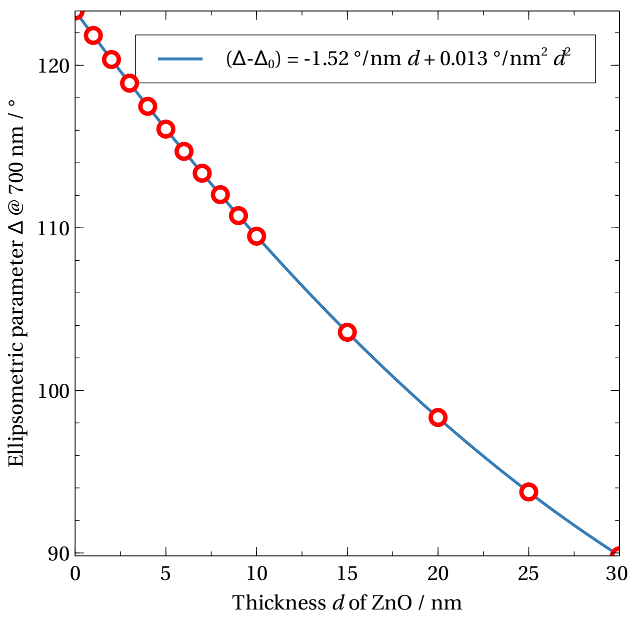

Figure S7: Dependence of ellipsometric parameter Δ at a wavelength of 700 nm on the thickness of ZnO for the system water–ZnO–Zn.

References

1. Zuo, J.; Erbe, A. *Phys. Chem. Chem. Phys.* **2010**, *12*, 11467–11476. doi:10.1039/c004532b.

2. Chen, Y.; Schneider, P.; Liu, B.-J.; Borodin, S.; Ren, B.; Erbe, A. *Phys. Chem. Chem. Phys.* **2013**, *15*, 9812–9822. doi:10.1039/C3CP44714F.

3. Chen, Y.; Schneider, P.; Erbe, A. *Phys. Status Solidi A* **2012**, *209*, 846–853. doi:10.1002/pssa.201100542.

4. Chen, Y.; Erbe, A. *Surf. Sci.* **2013**, *607*, 39–46. doi:10.1016/j.susc.2012.08.006.

5. Hans, M.; Erbe, A.; Mathews, S.; Chen, Y.; Solioz, M.; Mücklich, F. *Langmuir* **2013**, *29*, 16160–16166. doi:10.1021/la404091z.

6. Toparli, C.; Sarfraz, A.; Erbe, A. *Phys. Chem. Chem. Phys.* **2015**, *17*, 31670–31679. doi:10.1039/C5CP05172J.

7. Toparli, C.; Sarfraz, A.; Wieck, A. D.; Rohwerder, M.; Erbe, A. *Electrochim. Acta* **2017**, *236*, 104 –115. doi:10.1016/j.electacta.2017.03.137.

8. Toparli, C.; Hieke, S. W.; Altin, A.; Kasian, O.; Scheu, C.; Erbe, A. *J. Electrochem. Soc.* **2017**, *164*, H734–H742. doi:10.1149/2.0351712jes.

9. *Handbook of Ellipsometry*; Tompkins, H. G., Irene, E. A., Eds.; Willam Andrew Publishing - Springer Verlag: Norwich, Heidelberg, 2005.

10. Auinger, M.; Ebbinghaus, P.; Blümich, A.; Erbe, A. *J. Eur. Opt. Soc. Rapid Publ.* **2014**, *9*, 14004. doi:10.2971/jeos.2014.14004.

11. *Handbook of Chemistry and Physics*, 90th ed.; Lide, D. R., Ed.; CRC Press: Boca Raton, FL, USA, 2009.

12. Washington, P. L.; Ong, H. C.; Dai, J. Y.; Chang, R. P. H. *Appl. Phys. Lett.* **1998**, *72*, 3261–3263. doi:10.1063/1.121617.
